# Topology of the C-Terminal Tail of HIV-1 gp41: Differential Exposure of the Kennedy Epitope on Cell and Viral Membranes

**DOI:** 10.1371/journal.pone.0015261

**Published:** 2010-12-07

**Authors:** Jonathan D. Steckbeck, Chengqun Sun, Timothy J. Sturgeon, Ronald C. Montelaro

**Affiliations:** 1 Center for Vaccine Research, University of Pittsburgh, Pittsburgh, Pennsylvania, United States of America; 2 Department of Microbiology and Molecular Genetics, University of Pittsburgh School of Medicine, Pittsburgh, Pennsylvania, United States of America; University of South Florida College of Medicine, United States of America

## Abstract

The C-terminal tail (CTT) of the HIV-1 gp41 envelope (Env) protein is increasingly recognized as an important determinant of Env structure and functional properties, including fusogenicity and antigenicity. While the CTT has been commonly referred to as the “intracytoplasmic domain” based on the assumption of an exclusive localization inside the membrane lipid bilayer, early antigenicity studies and recent biochemical analyses have produced a credible case for surface exposure of specific CTT sequences, including the classical “Kennedy epitope” (KE) of gp41, leading to an alternative model of gp41 topology with multiple membrane-spanning domains. The current study was designed to test these conflicting models of CTT topology by characterizing the exposure of native CTT sequences and substituted VSV-G epitope tags in cell- and virion-associated Env to reference monoclonal antibodies (MAbs). Surface staining and FACS analysis of intact, Env-expressing cells demonstrated that the KE is accessible to binding by MAbs directed to both an inserted VSV-G epitope tag and the native KE sequence. Importantly, the VSV-G tag was only reactive when inserted into the KE; no reactivity was observed in cells expressing Env with the VSV-G tag inserted into the LLP2 domain. In contrast to cell-surface expressed Env, no binding of KE-directed MAbs was observed to Env on the surface of intact virions using either immune precipitation or surface plasmon resonance spectroscopy. These data indicate apparently distinct CTT topologies for virion- and cell-associated Env species and add to the case for a reconsideration of CTT topology that is more complex than currently envisioned.

## Introduction

Human immunodeficiency virus (HIV) infects humans predominantly through interaction of the viral envelope glycoprotein (Env) with the primary receptor CD4 and coreceptors CCR5 or CXCR4 on the surface of target cells. Env is produced as a 160 kDa polyprotein that is subsequently processed by extensive glycosylation, multimerization, and proteolytic cleavage to yield the virion-associated trimeric complexes of non-covalently associated gp120-gp41 dimers [1], [2]. Numerous studies have identified Env as a primary determinant of viral phenotypes; variations in Env sequence can affect cellular tropism, viral replication levels, immune recognition, and pathogenesis [1], [2]. Additionally, Env sequence variation has recently been experimentally demonstrated to be a primary determinant of lentivirus vaccine efficacy [3]. The majority of Env structural studies have focused on gp120 and the ectodomain of gp41; there is to date no definitive structural information on the approximately 150 amino acid long C-terminal tail that follows the proposed membrane-spanning domain (MSD) of gp41.

**Figure 1:**
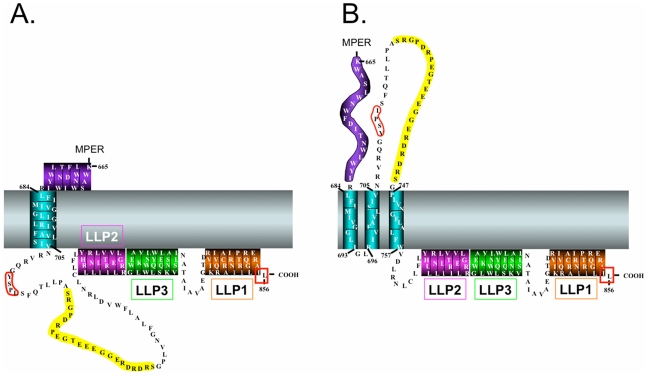
Schematic models of the HIV-1 CTT. A.) Traditional CTT model with one membrane-spanning α-helix and a completely intracytoplasmic localization of the remaining CTT sequence. LLP domains have been placed at their presumed membrane-localized position. B.) Alternative CTT model with multiple MSD segments as proposed by Hollier and Dimmock [15]. This model proposes three membrane-spanning β-sheets and an extracellular localization of the KE.

Studies addressing the CTT have traditionally examined: (i) the role of the CTT in viral Env incorporation [4], [5], [6], [7]; (ii) the influence of the CTT on virion maturation [4], [8], [9]; and (iii) the function of predicted endocytic signals present in the CTT [10], [11]. Various studies of the interactions of both cellular and other viral proteins as intracellular partners with Env have implicitly reinforced the traditional model of CTT topology as being localized completely within the inner surface of the cell or viral lipid membrane (Figure 1A).

Early evidence for an alternative topological model for the CTT was provided by Kennedy and colleagues [12], [13] who first reported that antiserum produced against a synthetic peptide from gp41 (residues 728–745) bound to HIV-1 Env, and that serum from HIV-1-infected humans also recognized this synthetic peptide [12]. Importantly, this group subsequently reported that antiserum raised against this synthetic peptide could specifically neutralize HIV in vitro [13]. These observations indicated exposure of the Kennedy epitope (KE) on the virion surface to allow antibody binding and neutralization, in direct contrast to the presumed intracytoplasmic location of the entire C-terminal sequences of gp41 following the MSD.

More recently, Dimmock and colleagues have attempted to address this apparent discrepancy between the traditional model of an exclusively intracytoplasmic CTT and an alternative model where the KE is exposed [14], [15]. Using antibodies directed to the 739ERDRD743 sequence and MAbs directed to the upstream 727PDRPEG732 and 733IEEE736 sequences in the KE, Dimmock and colleagues demonstrated virion binding and viral neutralization that was abrogated after pre-exposing virions to proteases [14]. Subsequent experiments using a MAb, SAR1, directed to 739ERDRD743 demonstrated that SAR1 effectively neutralized HIV-1 by apparently post-attachment binding at lower temperatures; negligible neutralization was observed when virus and cells were pre-incubated at 37°C [16], [17]. SAR1 was also shown to effectively inhibit virus replication in HIV-1 infected cells incubated in the presence of SAR1 antibody [16], likely through a decrease in cell-cell spread mediated by SAR1 inhibition of HIV-1 gp41-induced cell-cell fusion [18]. On the basis of these antibody accessibility studies, Dimmock and colleagues have proposed an alternative model for the topology of the HIV-1 gp41 CTT that predicts multiple MSDs and surface loops (Figure 1B) [14], [15].

This multiple MSD topology model is compatible with various studies that have identified intracellular partners proposed to interact with CTT sequences [6], [10], [19], [20], [21], but is incompatible with recent data indicating a requirement for both the Tyr-based sorting signal 711GYSPL715 and the extreme C-terminal dileucine sorting signal (boxed in red in Figure 1) in the CTT to facilitate Env endocytosis from the cell surface [11]. [10], [22].

Finally, more recent studies have examined the accessibility of the lentivirus lytic peptide (LLP) regions of the CTT (c.f. Figure 1) to antibody on Env-expressing cells [23]. Under native conditions, cell surface Env was not reactive with LLP1 and LLP2-specific antibodies, consistent with a sequestered localization of the LLP1 and LLP2 sequences [24]. Interestingly, however, when Env-expressing cells were incubated with CD4- and CCR5-expressing target cells at 31.5°C, binding of LLP2-specific antibodies was observed [23]. LLP2-specific MAbs did not bind when effector and target cells were incubated at 37°C, indicating a highly transient exposure of the LLP2 sequence [23], consistent with prior reports of LLP2 membrane association post-fusion [24]. These results indicate the need to reconsider the current static model of CTT topology and suggest that the CTT may be a more dynamic structure than previously thought.

**Figure 2:**
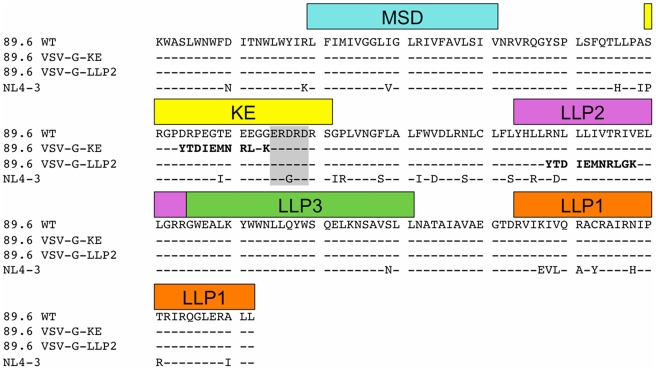
Sequence alignment of variant Env CTT. Sequence alignment of the membrane-spanning domain (MSD) and CTT of the Env proteins used in this study. All Env proteins were full-length gp160, however, only the sequences from K665 to the C-terminus are shown for simplicity. Structural and sequence domains are indicated in boxes above the corresponding sequence. VSV-G epitope tag indicated in bold; SAR1 and 1577 epitope indicated in gray box. KE – Kennedy epitope; LLP – lentivirus lytic peptide, MSD – membrane spanning domain.

In the current study, we sought to test the proposed topology models by experimentally determining the surface exposure of the KE sequences of gp41 for comparison to the external and internal locations predicted by the respective CTT models. Towards this goal, we have examined the accessibility of the KE to antibody binding on the surface of Env-expressing cells and on the surface of intact mature virions using a combination of flow cytometry, immunoprecipitation, and surface plasmon resonance (SPR) spectroscopy techniques that minimize the potential for disruption of the lipid membrane, the integrity of which is crucial to topology mapping studies (see Figure 2 for CTT sequences used). Our results indicate that the KE is topologically distinct on the cell surface as compared to the virion and that the topology of the CTT may be more complex and dynamic than indicated by current Env structural models.

## Results

### Exposure of CTT sequences on the surface of Env-transfected cells

To experimentally determine the exposure of the KE on the surface of Env-expressing cells, the antibody reactivity of transfected cells expressing HIV-1 89.6 Env with the VSV-G tag substituted in the KE (VSV-G-KE) or LLP2 (VSV-G-LLP2) segments was analyzed. The selected epitope allowed the use of a high affinity commercial anti-VSV-G MAb to compare the accessibility of a common epitope in the context of the KE segment and in a putative internal LLP2 segment of the CTT [23], [24]. Cells transfected with VSV-G-KE or VSV-G-LLP2 were compared to cells transfected with 89.6 WT following staining with anti-VSV-G and anti-actin antibodies. Anti-actin was used as a control for cellular membrane integrity, providing the ability to distinguish VSV-G positive intact cells from cells with compromised membranes.

**Figure 3:**
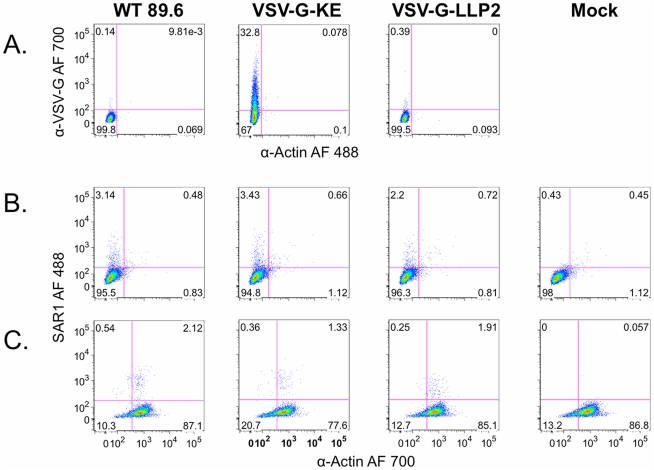
FACS analysis of intact Env-expressing cells demonstrates extracellular exposure of the KE sequence. Cells transfected with HIV-1 89.6 WT, VSV-G-KE, or VSV-G-LLP2 were analyzed by FACS to determine VSV-G epitope accessibility to anti-VSV-G MAb. A.) Intact, non-permeabilized Env-expressing cells were stained with α-VSV-G (AlexaFluor (AF) 700) and α-actin (AF 488). B.) Intact cells expressing Env were stained for surface exposure of the KE using a native KE antibody, SAR1. C.) Same as (B) except cells were permeabilized prior to staining.

As shown in Figure 3A, VSV-G-KE-expressing cells (middle) exhibit increased VSV-G-specific staining with no increase in actin staining compared to 89.6 WT (left) transfected cells, while VSV-G-LLP2-expressing cells (right) stain for neither VSV-G nor actin, similar to the control cells. Since the insertion of the VSV-G tag into the KE strategically conserved a key epitope for an existing KE-specific MAb (SAR1), cells expressing the various Env constructs were also tested for reactivity with this native sequence-specific MAb. All cells expressing Env show similarly increased SAR1 reactivity compared to mock-transfected cells without an increase in actin staining (Figure 3B). In addition to confirming the KE antibody accessibility results obtained with VSV-G-KE, this result also suggests that the observed lack of staining of 89.6 WT and VSV-G-LLP2 in Figure 3A is not due to decreased cell surface gp160 expression, as SAR1 reacted similarly with all Envs. Finally, to demonstrate the sensitivity of the actin stain to detect permeable membranes, SAR1 staining was repeated on permeabilized cells (Figure 3C). When the cell membrane integrity was compromised, anti-actin fluorescence intensity increased at least 10-fold, and 75–85% of permeabilized cells stained positive for actin. This result indicates that the lack of actin staining observed in Figure 3A and 3B reflects intact cell membranes and not poor actin reactivity.

**Figure 4:**
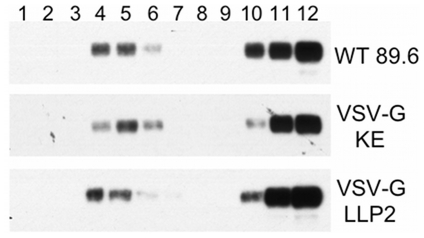
VSV-G epitope insertions do not disrupt Env association with detergent-resistant membranes. Env association with detergent resistant membranes was determined by sucrose gradient floatation followed by western blot analysis. Lane 1 represents the top of the gradient and lane 12 the bottom of the gradient. Blots were stained with anti-gp41 MAb Chessie 8 to determine the localization of gp41 in the bands shown.

Finally, as the CTT has been shown to contain signals important for association with detergent-resistant membranes (DRMs) [25], association of the Env constructs with detergent-resistant membranes (DRMs) was determined. Cells expressing 89.6 WT, VSV-G-KE, and VSV-G-LLP2 were subjected to membrane sucrose floatation gradients. As seen in Figure 4, all three Env constructs exhibited similar association with DRMs, suggesting that the VSV-G tag replacement strategy did not result in disruption of cell surface Env distribution. Taken together, these results indicate that the KE can be exposed on the surface of the plasma membrane, as the KE sequence is accessible to antibody binding in intact cells expressing Env, both in the context of the VSV-G epitope tag substitution and the native sequence.

### KE-specific antibodies do not bind to intact viral particles

Next, immunoprecipitation studies were performed to assess the ability of MAbs directed at the KE to pull down intact viral particles using protein G-coated paramagnetic beads. Both a gp120-specific antibody (termed C11) and a gp41 ectodomain specific antibody (7B2) were used to provide positive controls for Env binding and virion pull down. A p24-specific MAb (183-H12-5C) was used to detect disrupted virions in the pull down assays as a control for virion integrity. Of particular importance, several reference MAbs (2F5, 4E10, Z13e1) directed to the membrane proximal external region (MPER) of gp41 were used as controls, as this epitope is known to be closely associated with the membrane surface [26], [27], [28], [29]. Together these MAbs provided highly specific controls for antibody accessibility to viral membrane-associated epitopes, as predicted for the KE sequences in the Dimmock topology model (see Figure 1B). For intact virus immune precipitations, the level of antibody reactivity to its target antigen was measured by the relative level of viral p24 precipitated by the anti-Env MAbs (without lytic agents) compared to the amount of p24 (virus) input into the reaction.

**Figure 5:**
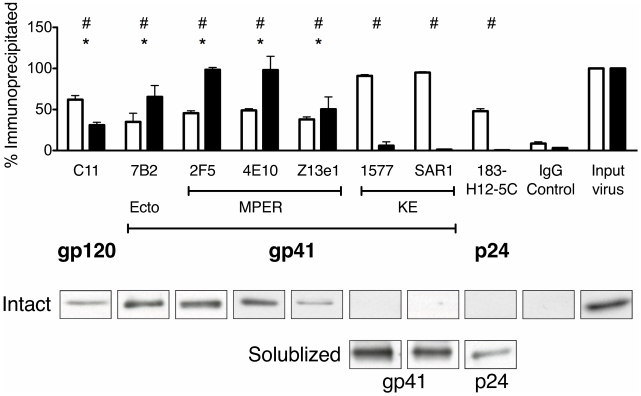
Anti-KE MAbs do not bind to intact virions. The indicated proteins and viral particles were immunoprecipitated using reference MAbs coupled to protein G-coated paramagnetic beads. (Top) Open bars represent % of target antigen precipitated when incubated with solubilized virus, while closed bars represent the % of input p24 precipitated by the corresponding MAb under native (intact virus) conditions. #  = p<0.05 for MAbs compared to IgG control with solubilized virus; *  = p<0.05 for MAbs with intact virus compared to IgG control. (Bottom) Representative p24 bands immunoprecipitated using the MAbs indicated in top panel with intact virus (Intact) or the bands of the target antigen from each MAb in detergent-disrupted virus (Solublized).

To ensure that all the reference MAbs were able to bind their target protein, immunoprecipitations were initially performed with MAbs incubated with purified virions solubilized with Triton X-100. The product immunoprecipitates were then characterized by SDS-PAGE and western blot for the respective target protein (e.g. C11 immunoblotted for gp120, 7B2 immunoblotted for gp41, etc.). The open bars in Figure 5 summarize the average results obtained for each MAb from three independent reactions with detergent-solubilized viral preparations. These data demonstrate that all of the reference MAbs effectively pulled down ≥50% of the total amount of target protein from solubilized virions; in contrast, the nonspecific IgG control precipitated less than 3% of the virion p24 indicating minimal nonspecific precipitation under these experimental conditions.

Having established that the MAbs were able to bind their target antigen in this assay format, we next tested the ability of the reference MAbs to precipitate intact virions under conditions similar to those used above without detergent solubilization. Results presented in Figure 5 (solid bars) demonstrate that only MAbs specific for gp120 (C11) or the known gp41 ectodomain epitopes (7B2, 2F5, 4E10, and z13e1) effectively pulled down the intact virus particles, as measured by p24 levels in the immunoprecipitate. In marked contrast, both KE-specific MAbs (1577 and SAR1) failed to immunoprecipitate virus (p24) to significant levels above the IgG control, indicating a lack of reactivity of these reference MAbs with the KE in the context of intact virions. Additionally, the p24-specific MAb (183-H12-5C) also failed to pull down p24 in intact virions, confirming the integrity of the virion preparation. Taken together, the results from these immunoprecipitation experiments indicate that the KE is not accessible to antibody binding in the context of intact viral particles.

To complement these immunoprecipitation studies, binding analyses of gp41-specific MAbs to purified virions in solution were performed using SPR spectroscopy. This technique allowed the assessment of qualitative differences in relative antibody affinity in real time during the course of the interaction. Specifically, SPR assays were used to address the possibility that both KE and MPER-specific MAbs bound viral particles equally well, but that KE MAb binding to virions was unstable during the washing procedures used in the immunoprecipitation assay.

**Figure 6:**
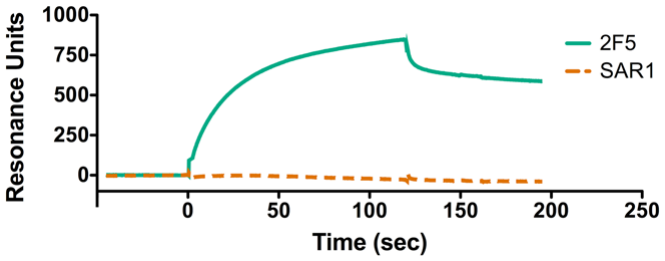
Comparison of MPER and KE MAb binding using SPR spectroscopy. MAbs 2F5 (MPER) and SAR1 (KE) were compared for relative binding rates and affinity using SPR spectroscopy to monitor antibody binding to purified intact HIV-1 virions. RU – resonance units.

As shown in Figure 6, the SPR results demonstrate stable binding of MAb 2F5 to the MPER of intact HIV-1 virions, but no detectable binding of SAR1 to the KE of gp41 in intact virions, despite the highly sensitive nature of this assay. These results are consistent with our viral immunoprecipitation observations and confirm that the KE is apparently inaccessible to antibody binding on the surface of viral particles and that the observed difference in KE accessibility in cells and viruses cannot be attributed to differences in the stability of antibody binding.

## Discussion

The current studies were designed to distinguish between the two distinct topological models currently proposed for HIV-1 gp41 by determining the accessibility of key CTT sequences in the context of virions and Env-expressing cells to binding by reference MAbs. The results of these studies for the first time reveal in parallel assays a marked difference in the accessibility to antibody binding of the KE in a primary-isolate Env expressed on the surface of cells compared to intact virions. Thus, these observations indicate that the CTT may exist in different topologies depending on the membrane environment, suggesting that the CTT may actually be dynamic in its association with different lipid membranes or other cofactors.

While the goal of this study was to distinguish between the alternative models of CTT topology, the current data is only partially compatible with both models. In the case of virion-associated Env, the lack of KE exposure to antibody binding is consistent with the classical model of Env topology (Figure 1A) in which the entire CTT sequence is sequestered within the lipid bilayer. In the case of Env expressed on the surface of cells, the accessibility of KE to antibody binding (and the inaccessibility of the LLP2) is consistent with an alternative Env topology (Figure 1B) in which parts of the CTT are exposed to the exterior of the lipid bilayer. To reconcile these two different models, it seems necessary to discard the static model of Env topology and to assume a new perspective in which CTT association with membranes is dynamic, perhaps controlled by interactions with other membrane binding proteins or lipid composition.

A dynamic model for CTT topology is consistent with the published studies by the Dimmock lab [16], [17] indicating that the KE is inaccessible to inactivation by SAR1 in free virions, but that the KE becomes exposed to SAR1 inactivation only after virion binding to target cells. The current data are also consistent with initial studies by Kennedy et al [12], [13] demonstrating that rabbit antiserum prepared against a KE synthetic peptide could inactivate HIV-1, as these early studies used an assay format in which the serum antibody was incubated with the virus and the target cells. Further supporting the concept of a dynamic CTT topology is the recent report indicating that the LLP2 domain of the CTT is only accessible to antibody during cell-cell membrane fusion, suggesting major changes in virion Env topology post receptor binding [23]. Interestingly, this would not be the first case of a viral envelope protein exhibiting a dynamic topology. The large envelope glycoprotein of hepatitis B virus is initially inserted into the endoplasmic reticulum membrane with its N-terminus located in the cytoplasm[30]. After a post-translational maturation process, the N-terminus is translocated across the membrane by insertion of another transmembrane helix in about 50% of the molecules [31]. It will be interesting and important to determine the extent and precise localization of KE-exposed Env on the cell surface.

Recent structural studies of diverse membrane proteins have resulted in an increased appreciation of the complexity and dynamics of membrane protein topology [30]. Earlier topology models were based on the simple concept that membrane proteins of the helix-bundle type were assembled from long hydrophobic alpha helices that were oriented perpendicular to the membrane plane [32]. However, based on high resolution structures of membrane proteins obtained during the past decade, it is now recognized that membrane helices can adopt a remarkable range of orientations relative to a membrane lipid bilayer and that membrane helices can dynamically reorient across the membrane in response to changes in the environment [30]. Specifically, a recent study has demonstrated that the six helix N-terminal domain of LacY can undergo topological inversion due solely to the introduction of phosphatidylethanolamine into the lipid membrane, illustrating that environmental changes can cause specific alterations in membrane protein topology [33]. Additionally, recent advances in understanding the properties of the lipid membrane have led to new insights into membrane protein-lipid interactions [34], [35]. In particular, hydrophobic matching of protein transmembrane segment length to lipid domains of similar thickness has been shown to affect membrane protein segregation, structure, and function [35], [36], [37], [38]. It is intriguing to consider, in the context of data presented here, the possibility that the gp41 CTT may undergo a dynamic change in topology due to alterations in the lipid environment. Alternatively, in order to minimize hydrophobic mismatch, Env with the CTT in the traditional topology could be selectively incorporated into the lipid rafts that serve as the sites of virion assembly.

A second aspect of current membrane protein structure studies that appears to be relevant to the case for a reconsideration of HIV-1 CTT membrane topology is the unique role of arginine (Arg) in membrane-associated proteins. While the early paradigm predicted Arg residues to be excluded from hydrophobic environments like a membrane lipid bilayer, more recent structural data indicate unique biochemical properties of Arg that produce unexpected functional properties for membrane proteins. For example, Arg residues can be incorporated as critical components of the membrane spanning domains of proteins, as observed with highly conserved Arg residue in the MSD of HIV-1 (c.f. Figure 1). The ability of the positively charged Arg to be located in a lipid environment is evidently due to the capacity of the Arg guanidinium group to form an energetically favorable cation-π bond with tryptophan, tyrosine, or phenylalanine residues in a stacked helix [39], [40]. Interestingly, a highly conserved Phe is located three residues C-terminal to the Arg in the HIV-1 MSD (Figure 2).

A second unique property of Arg is its ability in a repeat sequence (or homopeptide) to mediate transport of proteins and DNA or RNA across intact biological membranes, in contrast to homopeptides of other charged amino acids such as lysine (Lys) and histidine (His) that fail to efficiently cross lipid bilayers [41]. Thus membrane protein segments rich in Arg residues have a propensity to cross biological membranes under certain environmental conditions, offering the potential for a dynamic membrane topology. For example, the Arg-rich helical S4 segment of the voltage-gated potassium channel KvAP is proposed to move across the membrane as the channel opens and closes in response to changes in the membrane electric field [42]. While this was initially controversial due to the number of Arg residues (four) in the S4 segment, more recent studies have demonstrated that the S4 segment can insert into the membrane of eukaryotic cells as a transmembrane helix in spite of the four Arg residues [43]. These findings, when viewed in light of observations by Lu et al [23] demonstrating transient LLP2 exposure on Env-expressing cells undergoing fusion, may provide an explanation for the predominance of Arg residues in the HIV gp41 CTT relative to other cationic residues

A unique characteristic of the gp41 CTT of HIV-1 is that it contains two cationic amphipathic helical segments (LLP1 and LLP2) that are rich in Arg. In addition, the CTT contains a number of other highly conserved Arg residues. We and others have previously shown that synthetic LLP1 and LLP2 peptides assume a random coil conformation in aqueous environments, but rapidly convert to a helical conformation in a hydrophobic or membrane mimetic environment [44], [45], [46]. In addition, we have shown that synthetic LLP1 and LLP2 peptides are able to readily traverse biological membranes of intact mammalian cells. These observations are consistent with the concept of the Arg-rich CTT being intimately associated with the membrane lipids and being able to cross the lipid bilayer of an intact viral or cell membrane.

Finally, we and others have previously reported that modifications in CTT length or amino acid composition can have marked affects on global Env structure and function, including virion incorporation, fusogenicity, and antibody reactivity. For example, we found that the mutation of two Arg residues to glutamate (Glu) residues in LLP2 of an HIV-1 provirus substantially reduced Env fusogenicity and altered the antibody reactivity and neutralization sensitivity of epitopes located in both the gp120 protein and the ectodomain of gp41 [47]. These observations have been interpreted to indicate that changes in CTT result in allosteric alterations in global Env conformation that are reflected in altered functional properties, including reduced fusogenicity. It is interesting to speculate that altering the Arg content may affect the association of the LLP segments with the lipid bilayer and perhaps the ability of the CTT to traverse the membrane during the fusion process.

As often happens in HIV-1 research, our efforts to test two relatively simple models for CTT topology have indicated the need to consider more complicated and dynamic models of CTT membrane topology. This is perhaps a timely pursuit in light of the new paradigms of membrane structure and the importance of understanding the mechanisms by which the CTT serves as a determinant of HIV-1 Env structure and function on the surface of virions and cells.

## Materials and Methods

### Cells and virus preparations

HEK293T cells (ATCC) were maintained in DMEM (Invitrogen) supplemented with 10% (v/v) FBS. Cells were used at passage numbers less than 30. Gradient purified aldrithiol-2-inactivated HIV-1 NL4-3 virus (generously provided by Dr. Jeff Lifson, SAIC Frederick, Inc) used in this study has been described previously [48] and recently used for cryoelectron microscopy studies of virion-associated Env [49], [50], [51], [52]. The SAR1 hybridoma was a kind gift from Dr. Nigel Dimmock.

### VSV-G epitope tag substitutions

As described for topology mapping of other membrane proteins [53], [54], [55], we determined the antibody reactivity of selected Env constructs containing selected VSV-G epitope substitutions in the CTT. For these studies, the codon-optimized Env gene of HIV-1 (89.6) was cloned into a p2CI vector derived from PCR2.1 by insertion of the CMV promoter and polyA signal sequence from pcDNA3.1(hygro) (Invitrogen) and PCR-amplified IRES-Neomycin resistant sequence from pFB-Neo-LacZ vector (Stratagene). Overlapping PCR was used for the construction of the VSV-G substitution mutants of gp160. The two hybrid primers were constructed containing VSV-G tag sequences at the 5′-ends and HIV gp160 specific sequences at the 3′-ends of both the primers used for the substitutions. Final overlapping PCR products were then subcloned into the HIV-1 gp160 expression vector using the appropriate restriction enzymes and the Rapid DNA Ligation Kit (Roche Applied Science). All mutants were verified by DNA sequencing.

### Cell culture and transfections

HEK293T cells were plated into six-well plates 24 hours prior to transfection. 80% confluent cells were transfected with 2.5 µg of the selected HIV-1 Env mutant DNA using LipofectamineTM LTX reagent and PLUS™ reagent, as recommended by the manufacturer (Invitrogen).

### Cellular FACS analysis

Cells were harvested 24 hours post-transfection by treatment with 2 mM EDTA. Cells were resuspended and washed twice with FACS wash buffer (1X PBS with 5% FBS) at 4°C prior to antibody staining. Reference MAbs to VSV-G (Roche Diagnostics), gp41 (SAR1), and actin (Sigma-Aldrich) were labeled immediately prior to staining using Zenon labeling kits (Invitrogen) following manufacturer's instructions. 106 cells were dual-stained by incubating with 5 µg fluorophore-labeled antibody (VSV-G or gp41) and 7.5 µg fluorophore-labeled actin antibody for 30 minutes on ice. Following staining, cells were washed thrice with FACS wash buffer at 4°C. Washed cells were resuspended and stained with 7-amino-actinomycin D (7-AAD). Cells were not fixed prior to analysis. Fluorescently-labeled cells were analyzed on a FACSAria (BD Biosciences). Live, intact cells (7-AAD negative) were selected for scatter characteristics, including selection of single-cell populations by doublet-discrimination analysis. PMT settings were adjusted on identically-stained mock transfected cells prior to analysis of Env-transfected cells. Data was collected for 5×104 7-AAD negative cells and analyzed for reactivity with fluorescently-labeled VSV-G or gp41 and actin antibodies.

### Isolation of detergent resistant membranes

HEK293T cells were transfected with 89.6 WT, VSV-G-KE, or VSV-G-LLP2 plasmids. 48 hours post-transfection, cells were subjected to sucrose gradient floatation as previously described [56]. Briefly, cells were collected and washed in ice-cold 1X phosphate buffered saline (PBS). Washed cells were extracted on ice for 30 minutes with 0.5 ml ice-cold 1% Triton X-100 in NTE buffer (25 mM Tris-HCl; 0.15 M NaCL; 5 mM EDTA; pH 7.5) with phenylmethylsulfonyl fluoride and complete protease inhibitor (Roche). Cell lysates were centrifuged at 8,000×g for 10 minutes at 4°C. Clarified extracts were mixed with an equal volume of 85% sucrose in NTE and loaded into a prechilled centrifuge tube. Extracts were overlaid with 6 ml 30% sucrose in NTE, followed by 5 ml 5% sucrose in NTE. Tubes were centrifuged at 100,000×g for 18 hours at 4°C. The gradients were fractionated from the top manually by pipetting into ∼1 ml fractions. Aliquots of the samples were precipitated with trichloroacetic acid, resuspended in SDS-PAGE running buffer, and subjected to SDS-PAGE followed by western blot with anti-gp41 MAb Chessie 8. Blots were developed with PicoWest substrate (Pierce) and visualized on X-ray film.

### Viral immunoprecipitation and western blotting

Protein G Dynabeads (Invitrogen) were prepared according to the manufacturer's directions. Briefly, anti-Env or anti-Gag antibodies (4 µg) were incubated with 20 µl protein G Dynabeads in 35 µl citrate-phosphate buffer, pH 5.0, with gentle shaking for 45 minutes at room temperature. Isotype-matched IgG controls were used for each species (murine, human, etc.) from which a MAb was derived. Beads were washed thrice with 0.5 ml citrate-phosphate buffer followed by resuspension in either 26 µl PBS (for intact virus) or 26 ul PBS with 1% Triton X-100 (for lysed virus) and 4 µl NL4-3 virus (equivalent to 1.1 µg p24). Virus-bead suspensions were incubated at 4°C for one hour with gentle shaking and subsequently washed thrice with 1X PBS. Following the final wash, beads were resuspended in NuPAGE SDS-PAGE buffer, heated at 70°C for 10 minutes, and the supernatant loaded onto 4–12% Bis-Tris NuPAGE gels. Gels were electrophoresed followed by transfer to polyvinylidene fluoride (PVDF) membranes using the Invitrogen iBlot system. Blots were blocked for one hour in 5% blotto (1X PBS with 5% dry milk). After blocking, blots were cut to allow separate staining of gp120 (>60 kDa), gp41 (30–60 kDa), and p24 (<30 kDa). gp120 was stained with rabbit anti-gp120 (Advanced Biotechnologies, Inc.), gp41 stained with Chessie 8, and p24 stained with Ag3.0 for 1.5 hours at room temperature. Blots were washed thrice with 1X PBS and 0.025% Tween 20 (PBS-T), followed by incubation with appropriate secondary antibody (anti-rabbit IgG or anti-mouse IgG conjugated to horseradish peroxidase) for one hour at room temperature. Blots were washed thrice in PBS-T with the gp120 blot receiving an additional wash in 1X PBS with 0.1% Triton X-100. Finally, blots were incubated with PicoWest substrate (Pierce) for one minute and reassembled for visualization on X-ray film.

### Western blot quantitation

Antibody IPs were quantified by densitometry analysis. For each IP, three independent X-ray exposures were scanned and analyzed using ImageJ (NIH). For intact virus, p24 bands, and for solublized virus bands from each MAb's target antigen, were selected for each protein, and the integrated area under the densitometry curve was compared to that of the viral input band to yield percent of the input protein in the immunoprecipitate. Percent inputs for each protein for each of the three exposures were averaged to yield the overall percent input immunoprecipitated per experiment. This was repeated for three independent experiments, and the results were averaged to yield the final percent input immunoprecipitated per antibody. One-way ANOVA analysis was performed in Prism 5.0b with the Dunnett Multiple Comparison test and IgG as the control column. The P value of the test was <0.0001. Individual P values for antibodies compared to the IgG control are indicated in each data set.

### SPR spectroscopy analysis of antibody-virus interactions

Antibody-virus interactions were analyzed using SPR spectroscopy using a Biacore 3000 instrument. Protein A was immobilized on CM5 sensor chips as described [57], [58]. For analysis of the binding interaction, MAbs were captured on the protein A surface to ∼1500 RU with a protein A-only surface serving as a reference surface to account for virus-protein A non-specific interactions. Virus (2.5 mg/ml total protein; 172 µg/ml p24) diluted in 10 mM sodium phosphate buffer, pH 7.2 was injected over parallel reference and MAb-captured flowcells at 10 µl/min for 2 minutes. A buffer-only injection served to account for MAb dissociation from the protein A surface and was subtracted from each MAb binding experiment. Thus, presented sensorgrams are double referenced [59].
